# Upper Thoracic Spine Synovial Cyst Resulting in Paraplegia Following Transient Hypotension

**DOI:** 10.7759/cureus.9870

**Published:** 2020-08-19

**Authors:** Bradley T Schmidt, Andrea L Strayer, James A Stadler

**Affiliations:** 1 Neurosurgery, University of Wisconsin School of Medicine and Public Health, Madison, USA

**Keywords:** acute paraplegia, myelopathy, synovial cyst, thoracic spine

## Abstract

Development of synovial cysts in the rigid thoracic spine is rare. Additionally, synovial cysts with compression of nerve roots typically cause subacute or chronic radiculopathy. We present a patient who had a new diagnosis of upper thoracic (T1-2) synovial cyst that caused acute paraplegia while hospitalized for therapies and surgical planning.

The patient is a 56-year-old male with a history of congestive heart failure secondary to alcoholic cardiomyopathy. He presented with a progressive bilateral lower extremity discoordination, urinary incontinence, and altered perineal sensation. His examination revealed intact strength to bedside assessment, intact rectal tone, but upgoing toes on Babinski testing. Given concern for myelopathy, MRI thoracic spine was obtained and demonstrated large T1-2 synovial cyst causing severe compression with associated T2 signal change within the spinal cord. He underwent expedited cardiac optimization that included resumption of outpatient antihypertensive medications and the addition of a single dose of intravenous diuretic. The patient had subsequent transient hypotension following significant diuresis and developed acute paraplegia in his bilateral lower extremities. Fluids and vasopressors were initiated, and he underwent emergent surgery for decompression and synovial cyst resection. The patient did very well and had normalization of his neurological exam within 24 hours.

We present a case of acute paraplegia secondary to hypotension and spinal cord hypoperfusion in a patient with upper thoracic synovial cyst. This is rare pathology with an even more unique presentation. The authors recommend careful perioperative hemodynamic monitoring to help avoid acute worsening in this patient population.

## Introduction

Synovial cyst formation is common, particularly in mobile segments of the lumbar spine [1]. However, development of these cysts in the rigid thoracic spine is much more rare, with relatively few cases of this pathology reported [2-17]. Typically, synovial cysts arise at the facet joint and cause either lateral recess stenosis or exiting nerve root compression and subsequently cause subacute or chronic radiculopathy. We present a patient who had a new diagnosis of upper thoracic (T1-2) synovial cyst that was almost entirely medially projecting into the spinal canal and caused acute paraplegia while hospitalized for therapies and surgical planning. 

## Case presentation

The patient is a 56-year-old male with a history of congestive heart failure secondary to alcoholic cardiomyopathy. He initially presented with a progressive bilateral lower extremity discoordination, urinary incontinence, and altered perineal sensation. He had a history of low back pain that was exacerbated three weeks prior to presentation while shoveling snow. During this time, he experienced a subjective functional decline in his bilateral lower extremities with progressive difficulty walking. His pain radiated from his lower back to the posterior bilateral thighs, with the left worse than right. In addition, he endorsed urinary incontinence with uncontrolled dribbling of urine for a few days prior to his presentation. He denied any bowel function changes. Other than his cardiac history, he did not have other significant medical history; he does not smoke and has never had surgery on his spine previously. 

On initial evaluation, his exam was notable for full (5/5) strength in his bilateral upper and lower extremities, although he cannot bear his own weight on his legs. He had normal rectal tone, very slight subject loss of light touch to perineal region, normal (2+) reflexes at patella and achilles, and normal Hoffman testing. However, he did have abnormal Babinski testing bilaterally. 

**Figure 1:**
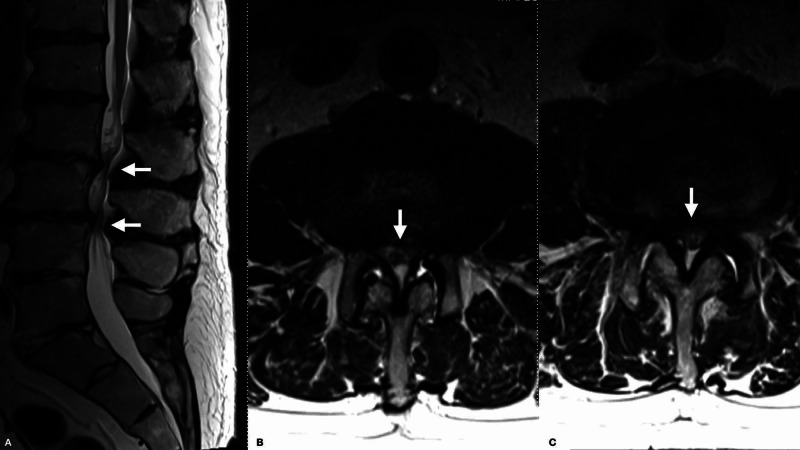
MRI lumbar spine demonstrating spinal stenosis at L2-3 and L3-4 Lumbar spine MRI demonstrating moderate to severe stenosis at L2-3 and L3-4 levels, see arrows identifying areas of severe stenosis. (A) Sagittal T2 image. (B) L2-3 level axial T2 image. (C) L3-4 level axial T2 image.

**Figure 2:**
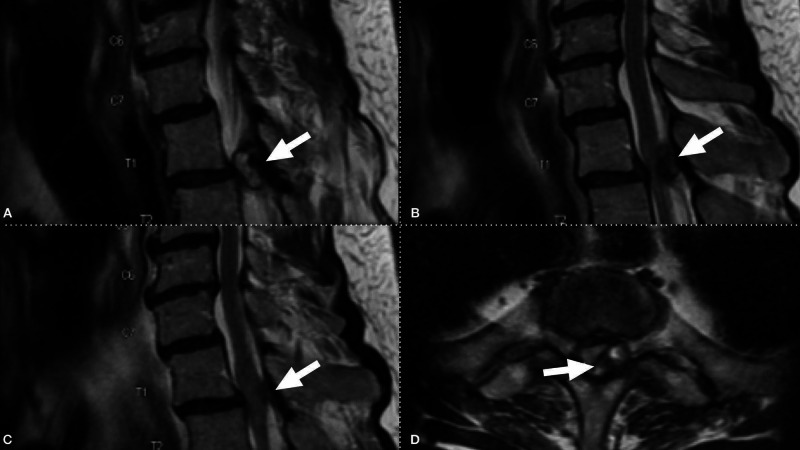
MRI of the thoracic spine demonstrating a large left T1-2 synovial cyst causing severe compression with associated T2 signal change within the spinal cord Cervical spine MRI demonstrating T1-2 synovial cyst, with severe spinal cord compression, see arrows demonstrating cyst and subsequent compression. (A,B) Sagittal T2 images demonstrating a large T1-2 synovial cyst causing spinal cord compression. (C) Midline sagittal T2 image demonstrating T2 signal changes within the spinal cord. (D) T1-2 level axial T2 images demonstrating severe compression from left-sided cyst, with the arrow demarcating the compressed and displaced spinal cord.

The patient was initially referred for neurosurgical evaluation given an MRI lumbar spine demonstrating spinal stenosis at L2-3 and L3-4 (Figure 1). However, the Babinski testing raised concern for myelopathy, and a cervical and thoracic spine MRI were obtained. The MRI of the cervical spine demonstrated mild degenerative changes. The MRI of the thoracic spine demonstrated a large left T1-2 synovial cyst causing severe compression with associated T2 signal change within the spinal cord (Figure 2). Given the clinical myelopathy, T2 signal changes on MRI, and the lack of radiculopathy or neurogenic claudication, the patient was offered a T1-2 laminectomy for synovial cyst resection prior to future consideration of lumbar decompression. Due to his reduced ejection fraction (25%-45%), he underwent expedited cardiac optimization, including the resumption of outpatient antihypertensive medications (lisinopril, carvedilol, and spironolactone) and the addition of a single 20 mg dose of intravenous furosemide. The patient had a subsequent transient hypotension following significant diuresis, with urine output of approximately three liters and blood pressure measured to 69/46 mmHg. Concurrently, he developed acute paraplegia with minimal to no movement at the left hip and knee and significantly decreased strength in the right lower extremity and left ankle (4- to 4/5). 

**Figure 3:**
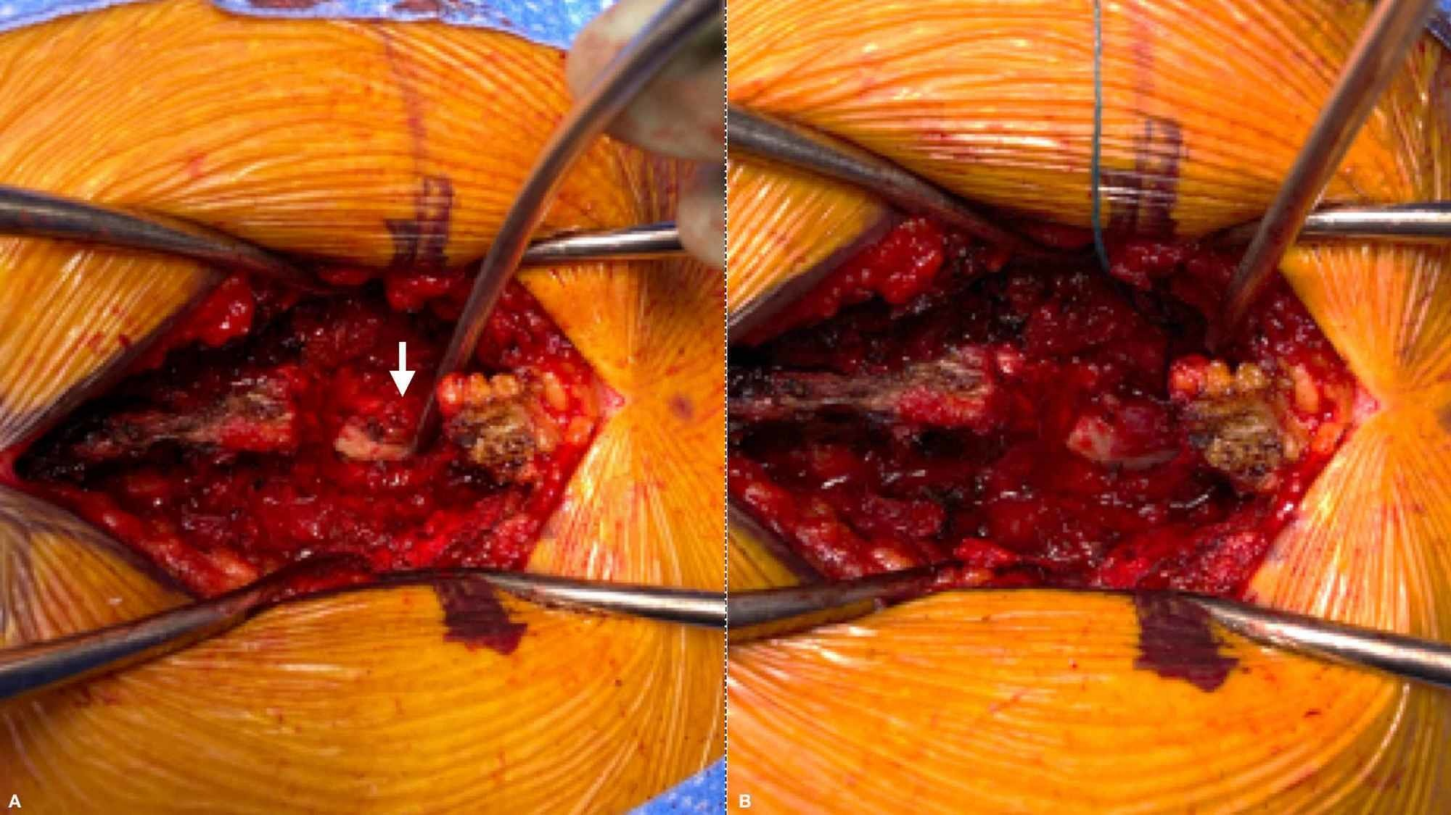
T1-2 laminectomy for spinal cord decompression and synovial cyst resection Intraoperative images demonstrating synovial cyst and subsequent resection. (A) Intraoperative image showing large synovial cyst after T1-2 laminectomy, note the cyst marked by the arrow. (B) Intraoperative image showing complete decompression of spinal cord following both laminectomy and cyst resection.

The patient was given a fluid bolus, phenylephrine infusion started, and he was taken emergently to the operating room for T1-2 laminectomy for spinal cord decompression and synovial cyst resection (Figure 3). Postoperatively his exam was notable for immediate partial improvement in his strength (left lower extremity grossly 3/5 throughout with 4/5 in foot dorsiflexion/plantarflexion) and consistent strength in right lower extremity. Vasopressor support was continued postoperatively for augmentation of his mean arterial pressure, and he had normalization of his neurological exam aside from the baseline myelopathic findings within 24 hours. He was discharged for further acute rehabilitation three days after surgery. 

## Discussion

In this report, we describe a patient with a large, compressive thoracic synovial cyst with sudden neurological deterioration associated with acute hypotension. Thoracic synovial cysts are rare, with a review of literature yielding only a few case reports [2-17]. Of the reports on thoracic synovial cysts, most are in the more mobile thoracolumbar junction [2-8,12]. Our case adds to the limited literature available on upper thoracic and cervicothoracic junction synovial cysts [9-15]. In one previously reported case, the cyst was also at T1-2, though the onset of symptoms in that case was subacute [15]. The synovial cyst in our case report originated from the left T1-2 facet joint and was medially projecting into the spinal canal rather than out the foramen. Upon review of the limited case reports available for these thoracic synovial cysts, many of them appear to be medially projecting into the canal as well [2,5,6,9,10,13-15,17]. We hypothesize that the coronally oriented nature of the thoracic spine facets may predispose synovial cyst formation in that trajectory rather than the more sagittally oriented facets of the lumbar spine, which may predispose cyst formation towards the lateral recess and foramen. Therefore, it is possible that synovial cysts of the thoracic spine may be at inherently higher risk for spinal cord compression and myelopathy. 

Importantly, this case also suggests that some patients may be at risk for sudden neurological decline despite the chronic nature of the underlying pathology. Symptoms related to spinal synovial cysts are typically gradual, though there are rare hyperacute presentations described in the literature, with one case of intracystic hemorrhage and one case of pain and weakness after direct blunt trauma [14,17]. Our patient had acute onset of lower extremity weakness and worsened myelopathy associated with hypotension, a result of inadvertently robust diuresis for preoperative cardiac optimization. While rare, a subset of patients may be more clinically tenuous and require careful hemodynamic management, a consideration that may not typically be considered in patients with chronic degenerative pathology.

Given the patient’s initial presentation with myelopathy and spinal cord compression, expedited surgery for resection of the large thoracic synovial cyst was initially planned. However, his normal strength exam allowed prioritization of medical risk optimization prior to surgery, an important consideration in patients with cardiac dysfunction. In this case, inadvertent hypotension during that optimization resulted a decline in strength likely secondary to spinal cord hypoperfusion. Based on this experience, for patients with significant neural element compression we recommend close neurological monitoring and avoidance of hypotension during preoperative cardiac risk assessment and management, as some patients may be more susceptible to sudden neurological decline despite the presumed chronic nature of synovial cysts.

## Conclusions

We present a case of acute paraplegia secondary to hypotension and spinal cord hypoperfusion in a patient with upper thoracic synovial cyst. This is rare pathology with a unique presentation, and this case therefore adds to the limited literature while demonstrating that some patients with this chronic degenerative finding may be at increased risk for sudden neurological decline. Careful perioperative hemodynamic monitoring may help avoid acute worsening, and emergent spinal cord decompression is appropriate when encountered.
